# Multi-Stage Temporal Convolutional Network with Moment Loss and Positional Encoding for Surgical Phase Recognition

**DOI:** 10.3390/diagnostics13010107

**Published:** 2022-12-29

**Authors:** Minyoung Park, Seungtaek Oh, Taikyeong Jeong, Sungwook Yu

**Affiliations:** 1 School of Electrical and Electronics Engineering, Chung-Ang University, 84 Heukseok-ro, Dongjak-gu, Seoul 06974, Republic of Korea; 2 School of Artificial Intelligence Convergence, Hallym University, Chuncheon 24252, Republic of Korea

**Keywords:** surgical phase recognition, Cholec80, moment loss, positional encoding, label smoothing, EfficientNet

## Abstract

In recent times, many studies concerning surgical video analysis are being conducted due to its growing importance in many medical applications. In particular, it is very important to be able to recognize the current surgical phase because the phase information can be utilized in various ways both during and after surgery. This paper proposes an efficient phase recognition network, called MomentNet, for cholecystectomy endoscopic videos. Unlike LSTM-based network, MomentNet is based on a multi-stage temporal convolutional network. Besides, to improve the phase prediction accuracy, the proposed method adopts a new loss function to supplement the general cross entropy loss function. The new loss function significantly improves the performance of the phase recognition network by constraining un-desirable phase transition and preventing over-segmentation. In addition, MomnetNet effectively applies positional encoding techniques, which are commonly applied in transformer architectures, to the multi-stage temporal convolution network. By using the positional encoding techniques, MomentNet can provide important temporal context, resulting in higher phase prediction accuracy. Furthermore, the MomentNet applies label smoothing technique to suppress overfitting and replaces the backbone network for feature extraction to further improve the network performance. As a result, the MomentNet achieves 92.31% accuracy in the phase recognition task with the Cholec80 dataset, which is 4.55% higher than that of the baseline architecture.

## 1. Introduction

The rapid development of artificial intelligence technology has influenced various fields of interest; more specifically, deep learning technology is being adopted in numerous ways in the field of medicine [1,2,3,4,5,6]. In particular, there is an increasing research focus on deep neural networks that process image data for various medical applications [7,8,9,10,11,12]. However, there are relatively few cases that focus on analyzing and utilizing medical videos (not still images) via deep learning techniques. This is mainly because it is quite complicated to apply deep learning techniques to video analysis since it is necessary to analyze not only each frame but also the correlation between frames.

The surgical video analysis algorithm can be described as the core of the medical context aware system (CAS), and it plays the following important roles [13,14,15]. First, during surgery, it monitors the status of the surgical procedure in real-time to detect emergencies such as excessive bleeding or abnormal surgical procedures to be able to prevent medical accidents in advance [16,17]. The medical CAS system can also aid the decision-making process of medical staff. In addition, it can be used to optimize operating room allocation and medical staff placement through surgical progress analysis [18,19,20]. For example, the CAS system can monitor the current surgical phase and make this information available for operation room resource management [21,22].

The CAS system is important not only during surgery but also after surgery. For example, the database indexing of surgical videos may be automated through the analysis of surgical videos, and the indexed surgical videos may be used to train unskilled surgeons and evaluate surgical skills [23,24,25]. In addition, the indexed surgical videos may also be utilized to analyze statistical information and optimize surgical workflows [14,26].

In view of this, Twinanda et al. proposed EndoNet, a network model that handles the phase recognition task in two steps. They also released the Cholec80 dataset to verify their model, which consists of 80 cholecystectomy surgery videos recorded at the speed of 25 fps [22]. In the first step of EndoNet, each frame is analyzed independently by using AlexNet [27] as the backbone architecture. However, the adjacent frames in a video are temporally correlated. This means that phase prediction networks should efficiently utilize this extra information for performance improvement. Thus, in the second step of EndoNet, a two-level hierarchical hidden Markov model (HMM) is adopted to exploit the correlation between different frames [22]. However, the HMMs assumes that the current state depends only on the previous states. More so, the number of states is limited to the number of classes defined in the problem.

To overcome this problem, Twinanda et al. published another paper in 2016, where they used a long short-term memory (LSTM) network (instead of the hierarchical HMM) to exploit the correlation information more efficiently [28]. By using the LSTM-based network, they could improve the prediction performance.

Many studies after the EndoNet-LSTM model have adopted a similar approach. For example, the SV-RCNet model in [13] also consists of two steps, where a feature extraction network is used in the first step and an LSTM network is used in the second step. More precisely, in the first step of SV-RCNet, ResNet [29] is used as the backbone architecture for feature extraction. Next, the extracted feature vectors are fed to the LSTM, which predicts the probability for each phase. The SV-RCNet used a down-sampled version of the feature vector sequence for end-to-end training. The MTRCNet is another example where the LSTM is adopted to exploit the temporal correlation information [17]. To improve the prediction performance, the MTRCNet uses a new loss component that makes use of the correlation between tool prediction and phase prediction.

Although LSTM-based networks could improve the performance of the phase recognition task, there are several drawbacks. First, the computational speed decreases significantly as the sequence length increases because LSTM-based networks process data sequentially. In addition, it is difficult to feed the whole video data to an LSTM at once. For example, most videos in the Cholec80 dataset are over 30 min long, and even if they are sampled at one frame per second (not at the original speed of 25 frames per second), they are still too large to be fed to an LSTM as a whole. Thus, the input sequence is usually divided into shorter (same-length) sequences before being fed to an LSTM. However, this procedure prevents the network from having a larger temporal receptive field, which, in turn, limits the performance improvement of the phase recognition network.

To solve this problem, TeCNO [30] took a different approach by adopting the idea of a multi-stage temporal convolutional network (MS-TCN) [31]. The MS-TCN is a popular method in the field of action segmentation and the core idea of MS-TCN is to introduce atrous convolution (i.e., dilated convolution) on the phase decision network. Due to the parallel characteristics of the atrous convolution, TeCNO could greatly reduce the computational cost. Additionally, TeCNO has a significantly wider receptive field than LSTM-based networks. As a result, TeCNO greatly improved the prediction performance and outperformed all the previous LSTM-based methods.

This paper proposes an improved phase prediction network. The proposed method also adopts the MS-TCN structure, but it exploits the inherent property of the Cholec80 surgery videos more efficiently than TeCNO. In addition, the proposed method makes use of the positional encoding [32,33,34,35] method, which is usually adopted in the transformer [32,33,36,37,38,39,40] applications. To the best of our knowledge, the proposed MomentNet is the first one to adopt the positional encoding technique in the MS-TCN structure. The main contributions of this paper are as follows:

1. Moment loss, which increases phase prediction accuracy by penalizing undesirable phase transition and preventing over-segmentation.
2. Positional encoding technique, which aids the network in figuring out contextual relations, which also improves the accuracy of the performance.

In addition, the proposed method applies a label smoothing technique to suppress overfitting and consequently, prevent the network from becoming over-confident. Overall, the techniques mentioned above, together with the replacement of feature extraction network, greatly improves the phase prediction accuracy. As a result, MomentNet shows significantly better prediction performance than the baseline architecture on the phase recognition task with the Cholec80 dataset.

## 2. Materials and Methods

**Figure 1:**
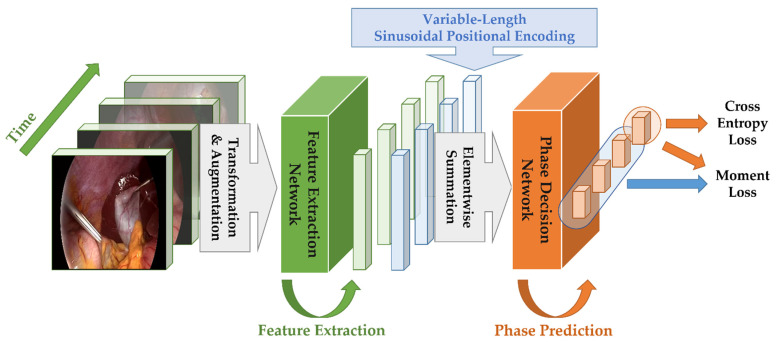
Overall block diagram of the proposed method.

Figure 1 shows the overall block diagram of the proposed method. Consistent with most conventional phase recognition networks, the proposed architecture also consists of two parts, where the first part is for feature extraction, and the second part is for phase decision. In the feature extractor, each video in Cholec80 dataset is sub-sampled at the speed of 1 frame per second. Then, each frame is fed to the feature extraction network. Following this, the extracted feature vectors (not the frames themselves) are fed to the phase decision network.

**Figure 2:**
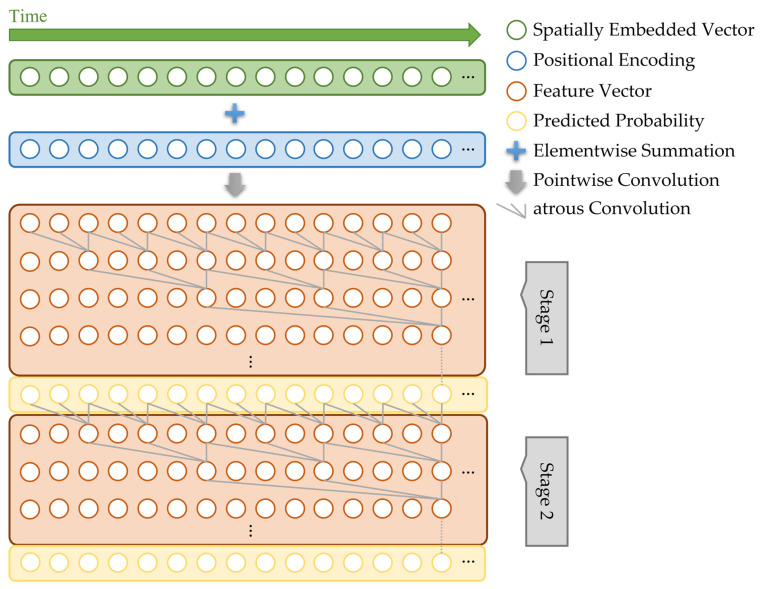
The structure of the proposed phase decision network.

As previously mentioned, the phase decision network of the proposed method is based on the MS-TCN. Figure 2 shows the overall architecture of the proposed phase decision network. Specifically, there are two stages in the MS-TCN of the proposed method, where each stage consists of several layers (For simplification, Figure 2 shows only 3 layers per each stage, but we use 8 layers in the proposed MomentNet). The spatially embedded vectors in Figure 2 represent the feature vectors from the feature extraction network in Figure 1. These input vectors are used as the input to the first stage, and the intermediate phase prediction results are obtained after the first stage. Then, these phase prediction results are refined in the second stage to produce the final phase prediction results.

**Figure 3:**
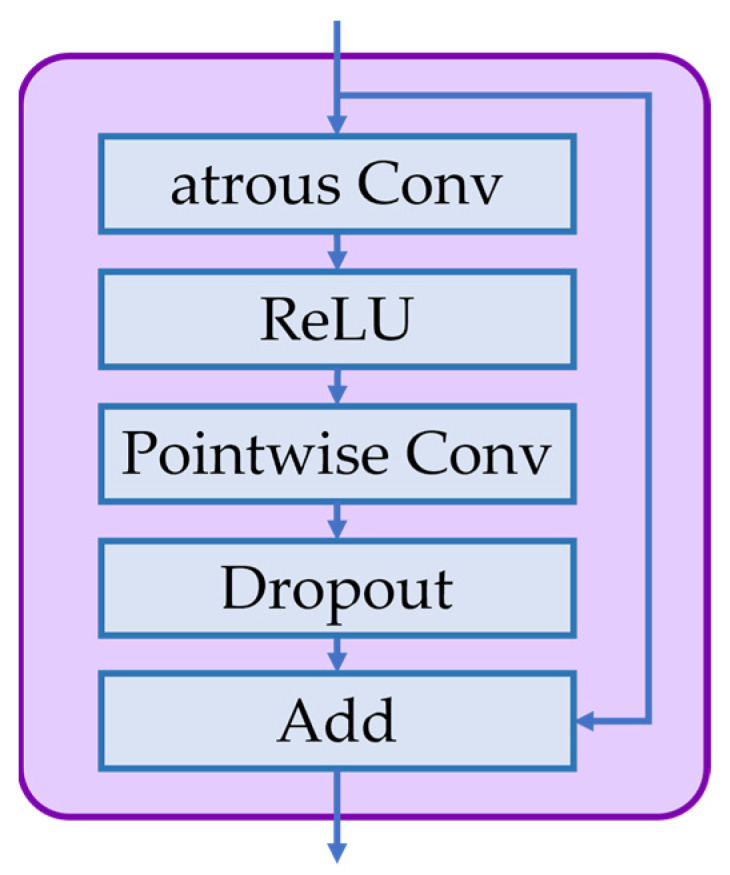
The operations performed in each layer of the phase decision network.

Figure 3 shows the operations performed in each layer of the phase decision network. As shown, each layer consists of atrous convolution (i.e., dilated convolution), ReLU, pointwise convolution (i.e., 1 × 1 × 1 convolution), and dropout operation. The following equations show these operations in more detail.

(1) $$z^{l}=ReLU\left(W_{atrous}^{l}*\;X^{l-1}+b_{atrous}^{l}\right)$$

(2) $$X^{l}=D\left(W_{pointwise}^{l}*\;z^{l}+b_{pointwise}^{l}\right)+X^{l-1}$$

In Equations (1) and (2), $X^{l-1}$ and $X^{l}$ denote the input and output vectors of the l-th layer, respectively, and the operator (*) represents the convolution operation. The learnable parameters $W_{atrous}^{l}$, $b_{atrous}^{l}$, $W_{pointwise}^{l}$ and $b_{pointwise}^{l}$ denote the weight and bias parameters of the atrous convolution and pointwise convolution, respectively. The function D means the dropout operation [41] with the dropout probability of 0.5.

In the MS-TCN, the receptive field size can be made very large by using an increasingly larger dilation factor in the atrous convolution as the layer number increases. In MomentNet, the dilation factor for the *l*-th layer is given as follows, where *N* represents the total number of layers in each stage:

(3) $$Dilation\left(l\right)=2^{l-1},where\;l\;\in \left[1,N\right]\;$$

Then, assuming every kernel size of the atrous convolution layer is 3, the receptive field of a single stage with N layers is given as follows:

(4) $$Receptive\;Field\left(N\right)=2^{N+1}-1\;$$

It should be noted that this is a very large value compared to the receptive field used in the LSTM-based models [13,17]. Although it is possible to have a large receptive field (RF) value in the LSTM-based models, it would require huge computational cost due to the sequential nature of LSTM models. The MomentNet not only exploits these advantages of the MS-TCN, but also proposes several efficient techniques for further performance improvement, which will be explained in the following sub-sections.

### 2.1. Moment Loss

Typically, most phase recognition networks use the cross-entropy loss in the optimization process. The cross-entropy loss is certainly one of the most effective loss components, but we discovered that it does not reflect all the important characteristics of the Cholec80 videos. That is, the following observations should be considered to improve the performance of a phase recognition network.

First, the phase number does not decrease in most cases (i.e., it only increases or maintains a constant value). For example, the phase number does not change from 2 to 1, nor does it change from 4 to 3.

Second, the amount of phase change is usually 0 or 1. In other words, the phase number does not change abruptly from 1 to 5, nor does it change from 2 to 7.

Finally, the phase transition occurs only a very few times (In most cases, the phase transition occurs only 6 times because there are 7 phases, and the phase usually increases by 1).

It should be noted that this number (i.e., 6) is very small compared to the total number of frames. For example, when a video is 30 min long, and if we sample the video at the speed of 1 frame per second, there are 1800 frames. Thus, theoretically, the phase transition can occur 1799 times, but, in reality, it occurs only 6 times. It should be noted that there are a few exceptions in some of the videos. For example, some of the videos consist of only 6 phases (instead of the usual 7 phases), in which case the phase transition occurs only 5 times. In addition, the phase number sometimes decreases in some of the Cholec80 videos. However, in most cases, Cholec80 videos satisfy the above-mentioned three properties. Thus, a new loss component is required to reflect the following important observations.

(Obs1) The phase number does not decrease in most cases.

(Obs2) The amount of phase change is usually 0 or 1.

(Obs3) The phase change occurs only a very few times.

One simple way to reflect the above-mentioned observations would be to use the loss function of the following form:

(5) L(n)=|p(n)−y(n)| 

where p(n) is the final phase prediction and y(n) is the target class at time step *n*. However, p(n) can be obtained only after the arg-max operation, which is not appropriate in a loss function owing to the non-differentiable characteristics. To solve this problem, the proposed method introduces a new loss component called moment loss, which is based on the following (1st) moment of the phase.

(6) $$M\left(n\right)=\overset{C=7}{\underset{c=1}{\sum }}c\cdot \;p_{final}\left(n,c\right)\;$$

In Equation (6), $p_{final}\left(n,\;c\right)$ is the predicted probability that the phase is *c* at time step *n* at the final stage of the MS-TCN. The number *C* denotes the number of phases (i.e., classes), which is 7 for the Cholec80 dataset. Then, the proposed moment loss at time step *n* is given in the following three forms:

(7a) $$L_{mA}\left(n\right)=ReLU\left(-M\left(n\right)+\frac{1}{P}\sum_{k=n-P}^{n-1}M\left(k\right)\;\right),\;$$

(7b) $$L_{mB}\left(n\right)=ReLU\left(M\left(n\right)-\frac{1}{P}\sum_{k=n-P}^{n-1}M\left(k\right)\;\right),$$

(7c) $$L_{mC}\left(n\right)=\left|M\left(n\right)-\frac{1}{P}\sum_{k=n-P}^{n-1}M\left(k\right)\right|$$

In Equation (7), *P* is a hyperparameter that represents the number of past frames that should be examined to find out the recent phase tendency. There are two components in all three forms of Equation (7), where the first one is the moment at the current time step, and the second one is the average moment of the past *P* frames. First, let us consider the loss form in Equation (7a). There will be no loss when the current moment (i.e., M(n)) is larger than the (average) past moment, but a loss will occur when the current moment is smaller compared to the past moment. Thus, it is expected that $L_{mA}\left(n\right)$ will be effective in the sense of (Obs1). Furthermore, it should be noted that $L_{mA}\left(n\right)$ will be effective in the sense of (Obs3) as well because it suppresses excessive phase transition. Now, let us consider the loss form in Equation (7b). As can be expected, $L_{mB}\left(n\right)$ will be effective in the sense of (Obs2) and (Obs3). Finally, it can be seen that $L_{mC}\left(n\right)$ will be effective in the sense of all three observations. Although the three forms of moment loss can be used independently, they can be used in a combined way as follows.

(8) $$L_{moment}\left(n\right)={\;\lambda }_{1}L_{mA}\left(n\right)+{\;\lambda }_{2}L_{mB}\left(n\right)+{\;\lambda }_{3}L_{mC}\left(n\right)$$

Then, the total loss function for a whole video is given as follows:

(9) $$L_{total}=\frac{1}{N}\left(\overset{N}{\underset{n=1}{\sum }}L_{CE}\left(n\right)+\overset{N}{\underset{n=1}{\sum }}L_{moment}\left(n\right)\;\right)$$

where *N* is the total number of frames and $L_{CE}$ is the cross-entropy loss defined as follows:

(10) $$L_{CE}\left(n\right)=-\frac{1}{S}\overset{S}{\underset{s=1}{\sum }}\overset{C}{\underset{c=1}{\sum }}y\left(n,c\right)\log p\left(s,n,c\right)$$

In Equation (10), p(s,n,c) is the predicted probability that the phase is *c* at time step *n* for the MS-TCN stage whose stage number is *s*. The number *S* represents the number of stages, which is 2 in the proposed network. Thus, p(s,n,c) in Equation (10) is the same as $p_{final}\left(n,\;c\right)$ in Equation (6) when s=S. In Equation (10), the one-hot encoded label y(n,c) is given as follows, where *c* and *n* denote the class number and frame number, respectively:

(11) $$y\left(n,c\right)=\{\begin{array}{cc} 1 & \mathrm{when}\;c\;\mathrm{is}\;\mathrm{the}\;\mathrm{target}\;\mathrm{class}\;\;\mathrm{at}\;\mathrm{time}\;\mathrm{step}\;n\\ 0 & \mathrm{otherwise} \end{array}$$

It should be noted that both y(n,c) in Equation (11) and y(n) in Equation (5) represent the target class (i.e., target phase) at time step *n*. However, they are different in that y(n,c) is represented in one-hot fashion, whereas y(n) is not (In other words, y(n) is a single number). The effect of this new loss function will be discussed in more detail in Section 3.1.

### 2.2. Positional Encoding

Generally, most conventional phase recognition networks use LSTM-based networks to analyze the temporal correlation between adjacent frames. On the other hand, MomentNet uses a MS-TCN for the phase decision task. Although MS-TCNs have many merits over LSTMs, there are some disadvantages as well. For example, it is difficult for MS-TCNs to find out the order of the frames in a given video sequence because it processes all the input frames in parallel. This is in contrast to LSTMs, where the order of frames can be easily figured out because each input frame is fed to the decision network sequentially. To solve this problem, the proposed method adopts the positional encoding technique, which was first introduced for transformers [36]. To the best of our knowledge, this is the first attempt to apply a positional encoding technique to MS-TCN.

Equations (12) and (13) show the positional encoding vector for each (pos, d, i), where pos represents the vector position in a given sequence, *d* denotes the feature vector dimension, and i denotes the position index within a feature vector.

(12) $$PE_{pos,\;2i}=\sin\frac{pos}{Const^{\frac{2i}{d}}}$$

(13) $$PE_{pos,\;2i+1}=\cos\frac{pos}{Const^{\frac{2i}{d}}}$$

In Equations (12) and (13), Const is a constant that should be determined appropriately so that it can handle a very long input sequence (It is usually set to 10,000 in transformer applications). The positional encoding vectors are added to the feature vectors (that are the outputs from the feature extraction networks in Figure 1) to help the decision network figure out the order of the input frames.

**Figure 4:**
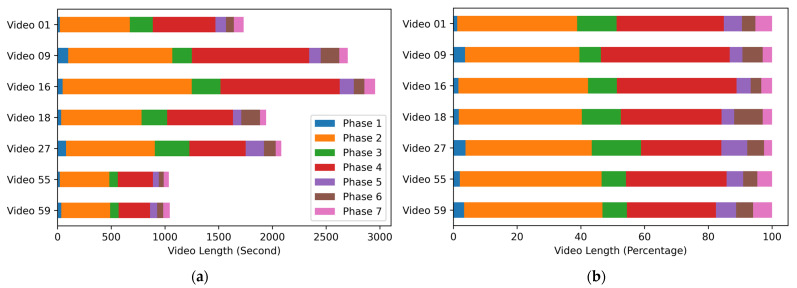
Phase transition points for sample videos in Cholec80 dataset: (**a**) Phase transition points shown in absolute time; (**b**) Phase transition points shown in relative time.

As will be shown in Section 3.2, this (basic) positional encoding technique improves performance to some extent, but the proposed method uses the technique in an even more efficient way. For this, we first investigated the characteristics of the Cholec80 dataset. The lengths of the 80 videos are quite different from one another, and the shortest video is only 739 s (12 min and 19 s) long, while the longest video is 5995 s (99 min and 55 s) long. As a result, the points at which the phase transitions occur are quite different for any two videos with significantly different video lengths. This can be seen in Figure 4a, where the phase transition points are shown in the unit of seconds for several sample videos in the Cholec80 dataset. However, if the phase transition points are shown in the unit of percentages, the transition points are quite similar for all the videos, as can be seen in Figure 4b. Thus, it can be expected that a relative positional encoding will be more effective.

One of the simplest ways to implement the relative positional encoding technique is to use the variable-length positional encoding (VLPE) method in [35]. The following equations show how the positional encoding vectors are generated by VLPE:

(14) $$PE_{pos,\;2i}\left(Video\;Length\right)=\sin\frac{pos}{Video\;Length^{\frac{2i}{d}}}$$

(15) $$PE_{pos,\;2i+1}\left(Video\;Length\right)=\cos\frac{pos}{Video\;Length^{\frac{2i}{d}}}$$

As can be seen, the VLPE uses the video sequence length in the denominators of Equations (14) and (15), whereas the basic PE uses a constant. Thus, the VLPE can be used only when the video length is known in advance, whereas the basic PE technique can be used without knowing it. The effect of this technique (including the comparison between basic PE and VLPE) will be shown in Section 3.2.

### 2.3. FeatureNet Backbone Architecture and Label Smoothing

Typically, most conventional phase recognition networks are based on the ResNet architecture, and TeCNO also uses ResNet50 for feature extraction. As the depth, width, or resolution of a CNN increases, the performance of the CNN improves, but, at the same time, the computational cost also increases. However, an absurdly large increase in any one of these factors will result in an exponentially expensive computational cost, with very little or no performance gain. Thus, it is important to determine these three factors (i.e., the depth, width, and resolution) in a balanced way for the best trade-off between performance and computational cost. To solve this problem, the EfficientNet [42] presents a scaling technique called the compound scaling method to find the optimal ratio between these three factors, which is given in the following equation.

(16) $$\mathrm{depth}={\;\alpha }^{\phi },\;\;\;\mathrm{width}=\beta^{\phi },\;\;\;resolution={\;\gamma }^{\phi },\;\;s.t.\alpha \cdot \beta^{2}\cdot \gamma^{2}\approx 2,\alpha \geq 1,\;\beta \geq 1,\;\gamma \geq 1$$

In Equation (16), α, β,γ are constants determined by a small grid search and the coefficient ϕ denotes a hyper-parameter that can control the model scaling. The proposed method chooses EfficientNetB4 as the backbone architecture for the feature extractor. As shown in Table 1, EfficientNetB4 requires a similar number of mult-adds operations as ResNet50, and it requires fewer parameters than ResNet50. However, EfficientNetB4 demonstrates a better performance than ResNet50, which will be discussed in more detail in Section 3.3.

In general, the higher the number of classes, the lower the prediction accuracy in multi-class classification tasks. However, in the phase recognition task with the Cholec80 dataset, the phase prediction accuracy is usually not high enough although there are only 7 classes. Since the prediction accuracy usually reaches 100% in the training stage, this implies that a phase recognition network does not generalize well. We investigated the reasons for this bad generalization and attributed this problem to the low variation between frames in the Cholec80 dataset. That is, most frames in a Cholec80 video share similar colors and objects and consequently, there are only small differences between frames. Thus, a phase recognition network should use this small difference as a clue to determine the current phase, which, in turn, causes over-fitting. Thus, we decided to apply label smoothing [43] to prevent the network from becoming over-confident and make the network generalize well with un-seen data. In conventional phase recognition networks, the target label is given in the one-hot fashion, as shown in Equation (11). On the other hand, in the MomentNet, label smoothing is applied as follows, where *C* is the number of classes and α is a smoothing parameter:

(17) $$y\left(n,c\right)=\{\begin{array}{cc} 1-\alpha & \mathrm{when}\;c\;\mathrm{is}\;\mathrm{the}\;\mathrm{target}\;\mathrm{class}\;\mathrm{at}\;\mathrm{time}\;\mathrm{step}\;n\\ \frac{\alpha }{C-1} & \mathrm{otherwise} \end{array}$$

The larger the α, the stronger the label smoothing effect. However, if α is too large, the difference between 1−α and α/(C−1) becomes too small, which, in turn, hinders the training process. Thus, it is very important to carefully determine the value of α. The optimal value of α and the effect of the label smoothing technique will be discussed in more detail in Section 3.3.

### 2.4. Training Configuration

**Figure 5:**
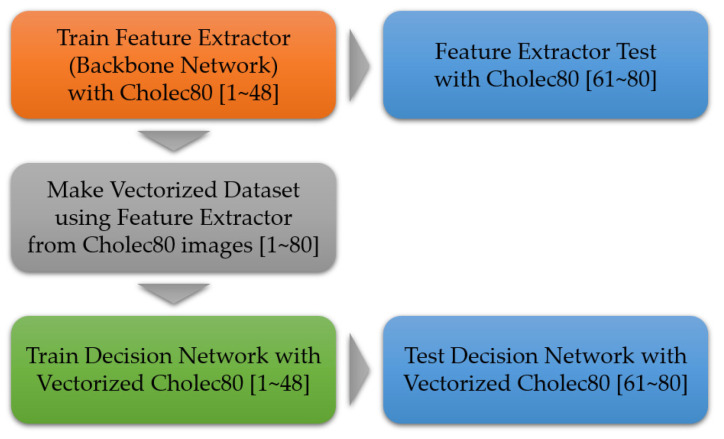
The training process of the proposed network.

Figure 5 shows the overall training process of the MomentNet. As mentioned in Section 1, the dataset that we used is Cholec80, which is the most popular public dataset in the phase recognition field. There are 80 videos in the Cholec80 dataset, and the train, validation and test dataset split ratio is 48:12:20. Each video in the Cholec80 dataset is sub-sampled at the speed of 1 frame per second. The overall training process consists of feature extractor training and decision network training.

In the first step, each frame (in the train split) is fed to the feature extraction network for training. To maximize the feature extractor performance, both tool detection information and phase recognition information were exploited by using the binary cross entropy loss function. For the feature extractor training, the sampled images were preprocessed via resizing to 350 by 350 pixels, random cropping to 320 by 320 pixels, random horizontal flipping with probability of 0.5, color jittering, and input normalization. As an optimizer, the AdamW [44] was adopted with a batch size of 8, a weight decay of 1E-2, and a start learning rate of 5E-4. Furthermore, as the learning rate scheduler, the cosine annealing method [45] was applied. Specifically, the learning rate was reduced to 1E-6 by the end of the 5th epoch, and subsequently, a fixed learning rate of 1E-6 was used for 5 more epochs. At the end of each training epoch, validation was performed using the validation set, and the validation loss and validation accuracy were recorded to check if there was any abnormality in the training process.

Then, in the second step, the vectorized feature dataset is built by using the pre-trained feature extractor network. In this step, the input images were preprocessed via resizing to 350 by 350 pixels, center cropping to 320 by 320 pixels, and input normalization.

Finally, the vectorized dataset was used to train the phase decision network. It should be noted that only the decision network is trained in this step (In other words, the learning parameters in the feature extractor are not updated in this step). In this step, the AdamW optimizer was used again as an optimizer, with a weight decay of 1E-2 and the start learning rate of 5E-4. The learning rate was reduced to 1E-6 until the 40th epoch by the cosine annealing method. The batch size is set to be equal to the length of each video.

## 3. Results

### 3.1. Effect of the Moment Loss Function

As explained in Section 2.1, three forms of moment loss can be used independently, or they can be used in combination as shown in Equation (8). The row numbers 3 to 5 in Table 2 show the accuracy improvement when each of the three forms is used independently. The baseline accuracy is based on the TeCNO model, which uses only the cross-entropy loss. The hyperparameter *P* in Equation (7) is set to 10 in this experiment. Although the value of *P* can be optimized for each case, one single value was used for convenience.

It was first observed that the value of λ has a very important effect on the final performance. As can be expected, when λ was very small, the results were not significantly different from the baseline network that uses only $L_{CE}$. On the other hand, when a very large λ value was used, the network did not converge. Thus, the parameters $\lambda_{1},{\;\lambda }_{2},$ and $\lambda_{3}$ were optimized separately for each of the three cases by using the grid search method. As can be observed, a performance improvement of at least 0.86% was observed when each of the three forms was used independently.

It is also observed that the result with $L_{mA}\left(n\right)$ is most accurate, which implies that (Obs1) and (Obs3) in Section 2.1 are relatively more important than (Obs2). It can also be seen that the optimal $\lambda_{1}$ is relatively larger than the optimal $\lambda_{2}$ and $\lambda_{3}$. This is because the events that violate (Obs1) happen less frequently compared to the events that violate (Obs2) or (Obs3) (Here, a violation of (Obs1) means that a predicted phase is smaller than the average past phase). Thus, a relatively larger value of $\lambda_{1}$ is required for appropriate training. As shown in the final row of Table 2, the best result was observed when the three forms were used in a combined way. In fact, the performance of MomentNet is 1.85% better than that of the baseline network, which is a significant improvement in performance.

### 3.2. Effect of (Variable-Length) Positional Encoding

Table 3 shows the effect of the positional encoding technique presented in Section 2.2. As shown in the table, both basic PE and VLPE techniques effectively improve the accuracy of the baseline model. It is also observed that VLPE is more effective than basic PE. More precisely, the accuracy of the proposed model based on PE is 0.53% higher than that of the baseline model, while the accuracy of VLPE model is 1.31% higher. This is because a relative position is a better position indicator when dealing with many videos with different lengths. It should be noted that the VLPE technique can be used only when the video length is known in advance. Thus, it can be used only for ‘after-surgery’ applications, not for ‘during-surgery’ applications. It should be noted that there are many ‘after-surgery’ applications as well as ‘during-surgery’ applications, as explained in Section 1. It should also be noted that the basic PE can be used for both cases.

### 3.3. Effect of Other Optimization Techniques

As explained in Section 2.3, EfficientNetB4 requires fewer parameters and approximately the same number of operations, when compared with ResNet50. However, a decent performance improvement was observed when EfficientNetB4 is used instead of ResNet50 as the backbone architecture of the feature extraction network. Table 4 shows the comparison results between the two backbone architectures (It should be noted that the moment loss and the PE techniques were not used here. The comprehensive results will be shown in Section 3.4). As can be seen, the accuracy with the EfficientNetB4 network is 0.85% higher than that of the ResNet50 case.

Table 4 also shows the effect of the label smoothing. As explained, when the label smoothing parameter α is very small, the performance improvement is negligible. On the other hand, as α increases, the accuracy begins to increase as well. However, when α becomes too large, the performance begins to degrade, as can be seen in Table 4. According to our simulations, the optimal α value was 0.20 for both ResNet50 and EfficientNetB4 backbone architectures.

### 3.4. Combined Results

Table 5 summarizes all the results shown in Section 3.1, Section 3.2 and Section 3.3. It also shows the combined results when all the techniques are used together. It should be noted that the effect of one technique can affect the effect of another one. As a result, the optimal λ values in the final two rows of Table 5 are slightly different from the ones shown in the third row. As shown, when the base PE is applied, the final accuracy of the MomentNet is 91.90%, which is 4.14% higher than that of the baseline architecture. On the other hand, when VLPE is used, the accuracy of the MomentNet is 4.55% higher, which is a significant improvement in performance. As explained, a proper PE technique should be used depending on the application being considered.

**Figure 6:**
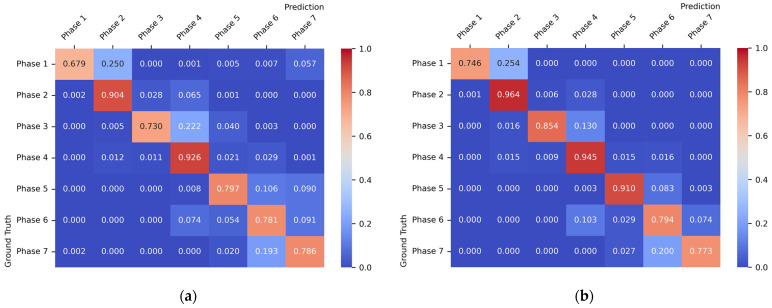
Confusion matrices for the baseline model and our proposed model. (**a**) Baseline architecture. (**b**) MomentNet.

Figure 6 compares the confusion matrix of the baseline network with that of MomentNet. The options shown in the last row of Table 5 were used for the confusion matrix results in Figure 6b. For convenience, let *p*(*i*, *j*) denote the probability that the predicted phase is *j*, while the ground truth label is *i*. Then, the following equation should hold for all *i*:

(18) $$\overset{7}{\underset{j=1}{\sum }}p\left(i,j\right)=1\;\mathrm{for}\;1\leq i\leq 7$$

From Figure 6, it is observed that the following property holds for the MomentNet case, whereas it does not hold for the baseline model.

(19) p(i,j)=0     when |i−j|≤3

This is mainly due to the moment loss, which penalizes the phases that are far from the target phase (i.e., target class). Let us give an example. Suppose that the target phase at the current time step *n* is 2. Then, among all $p_{final}\left(n,\;c\right)$ in Equation (6), $p_{final}\left(n,\;c=2\right)$ should be the highest (preferably with very large differences when compared with other $p_{final}\left(n,\;c\right)$ values). This means that M(n) in Equation (6) will be very close to 2. Then, considering the three observations in Section 2.1, (1/P)∑M(k) in Equation (7) will also be close to 2. Then, $p_{final}\left(n,\;c=7\right)$ in Equation (6) will be suppressed because a large $p_{final}\left(n,\;c=7\right)$ would increase the value of M(n), which, in turn, would increase the moment loss in Equation (7). Of course, $p_{final}\left(n,\;c=6\right)$ will also be suppressed in this example although the degree of suppression will be smaller than that for $p_{final}\left(n,\;c=7\right)$ case. It is also observed that *p*(*i*, *i*) of MomentNet is higher than that of the baseline model for every *i*. Although Figure 6b is based on the options shown in the last row of Table 5, a similar result was observed for a different option combination as long as the moment loss is used.

**Figure 7:**
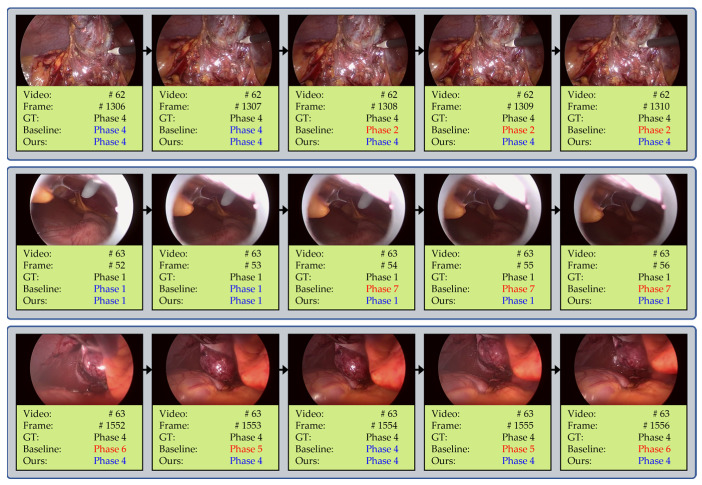
Test examples of the baseline model and our proposed model.

Figure 7 shows three examples where the proposed method makes correct predictions while the baseline method does not. More precisely speaking, the three figures in Figure 7 show examples where the baseline method fails to reflect (Obs1), (Obs2) and (Obs3) in Section 2.1, respectively. On the other hand, the MomentNet successfully makes correct predictions in the three cases by making use of the proposed moment loss.

## 4. Conclusions

Surgical video analysis algorithms are very important in many medical applications. In particular, phase recognition algorithms play very important roles both during and after surgery. This paper proposed an efficient phase recognition network, called MomentNet, for cholecystectomy endoscopic videos. To improve the performance of a phase recognition network, it is important to constrain unwanted phase transitions. Because of the non-differentiable characteristics of the argmax function, it is difficult to give a penalty for undesirable phase transitions. However, by proposing a novel idea, called moment loss, MomentNet successfully improved the phase recognition performance. It is also demonstrated that positional encoding can help improve performance. To the best of our knowledge, MomentNet is the first attempt to apply a positional encoding technique to the MS-TCN, although there have been very many papers that apply the positional encoding technique to transformer architectures. Although both basic PE and VLPE improved the phase prediction performance, VLPE was more effective because relative position is a better position indicator. The replacement of the feature extraction architecture also proved to be quite effective. Although EfficientNetB4 requires a similar number of operations and fewer parameters, when compared with ResNet50, the new backbone architecture was quite helpful in increasing the phase prediction accuracy. Finally, the label smoothing technique prevented MomentNet from becoming over-confident and it also improved the performance.

It should be noted that the three properties in Section 2.1 will also hold for most other surgery videos. This means that the proposed moment loss can be used not only for the Cholec80 dataset, but also for other surgery videos (although slight changes may be required). This is another merit of the proposed method, and this topic should be investigated further in future work. Finally, it should also be noted that action segmentation is another area where the MS-TCN structure can be efficiently used. Thus, the proposed moment loss and positional encoding idea may be applied to the action segmentation area, which is another candidate for future work.

## Figures and Tables

**Table 1:** Comparison between ResNet50 and EfficientNetB4.

	ResNet50	EfficientNetB4
output feature vector size	2048	1792
# of parameters	26 M	19 M
# of operations	4.1 B Flops	4.2 B Flops

**Table 2:** The accuracy improvement due to different combinations of three moment loss components.

	$\lambda_{1}$	$\lambda_{2}$	$\lambda_{3}$	Accuracy	Improvement
Baseline				87.76%	
$L_{mA}$ only	15			89.00%	1.24%
$L_{mB}$ only		7		88.62%	0.86%
$L_{mC}$ only			5	88.65%	0.89%
Combination	10		3	89.61%	1.85%

**Table 3:** The accuracy improvement due to (variable length) positional encoding.

	Accuracy	Improvement
Baseline Network	87.76%	
(Basic) Positional Encoding	88.29%	0.53%
Variable-Length Position Encoding	89.07%	1.31%

**Table 4:** Effect of other optimization techniques.

Feature Extractor	Label Smoothing	Accuracy	Improvement
ResNet50		87.76%	
	0.01	88.43%	0.67%
	0.05	88.98%	1.22%
	0.10	89.07%	1.31%
	0.20	89.20%	1.44%
	0.30	89.00%	1.24%
	0.40	88.97%	1.21%
EfficientNetB4		88.61%	0.85%
	0.01	89.87%	2.11%
	0.05	90.32%	2.56%
	0.10	90.54%	2.78%
	0.20	91.07%	3.31%
	0.30	90.94%	3.18%
	0.40	90.89%	3.13%

**Table 5:** Summary of all the results of MomentNet.

	Feature Extractor	Label Smoothing	Moment Loss	PE	Accuracy	Improvement
Single Technique	ResNet50				87.76%	
	ResNet50		$\left[\lambda_{1},\lambda_{2},\lambda_{3}\right]=\left[10,\;0,\;3\right]$		89.61%	1.85%
	ResNet50			Basic PE	88.29%	0.53%
	ResNet50			VLPE	89.07%	1.31%
	ResNet50	0.20			89.20%	1.44%
	EfficientNetB4				88.61%	0.85%
Combined Results	EfficientNetB4	0.20	$\left[\lambda_{1},\lambda_{2},\lambda_{3}\right]=\left[5,\;0,\;2\right]$	Basic PE	91.90%	4.14%
	EfficientNetB4	0.20	$\left[\lambda_{1},\lambda_{2},\lambda_{3}\right]=\left[5,\;0,\;2\right]$	VLPE	92.31%	4.55%
